# Molecular Characterization and Expression Pattern of *leptin* in Yellow Cheek Carp (*Elopichthys bambusa*) and Its Transcriptional Changes in Response to Fasting and Refeeding

**DOI:** 10.3390/biology12050758

**Published:** 2023-05-22

**Authors:** Min Xie, Jinwei Gao, Hao Wu, Xiaofei Cheng, Zhou Zhang, Rui Song, Shaoming Li, Jie Zhou, Cheng Li, Guoqing Zeng

**Affiliations:** 1 Hunan Fisheries Science Institute, Changsha 410153, China; xieminhaha@126.com (M.X.); gaojinwei163@163.com (J.G.); wh17380133463@163.com (H.W.); chengxiaofei19@126.com (X.C.); zz19961022@hotmail.com (Z.Z.); ryain1983@163.com (R.S.); licheng1969001@sina.com (C.L.); 2 College of Animal Science and Technology, Hunan Agricultural University, Changsha 410125, China; 17674042583@163.com; 3 Hunan Aquatic Foundation Seed Farm, Changsha 410153, China

**Keywords:** yellow cheek carp, *leptin*, gene structure, tissue expression, fasting and refeeding

## Abstract

**Simple Summary:**

This article is mainly about that molecular characterization and expression pattern of *leptin* in yellow cheek carp (*Elopichthys bambusa*) and its transcriptional changes in response to fasting and refeeding. In this study, the authors used PCR to clone the CDS of *leptin* in yellow cheek carp, and analyzed the sequence differences of the gene with other species, constructed the phylogenetic tree, used real-time PCR for analyzing the expression of *leptin* in different tissues, including the expression of *leptin* in the brain and liver after fasting–refeeding of yellow cheek carp. This paper found that the full-length cDNA sequence of *Eblep* was 1140 bp and the length of the open reading frame (ORF), which can encode a protein of 174 amino acids, was 525 bp. The Eblep mRNA transcript was detected in all tested tissues, with the highest expression in the liver and lowest expression in the spleen. It was found that the change in the mRNA expression of EbLep may be an adaptive strategy for different energy levels by studying the expression of EbLep mRNA in the brain and liver under fasting and refeeding.

**Abstract:**

*Leptin*, a secretory protein encoded by obese genes, plays an important role in regulating feeding and energy metabolism in fish. To study the structure and function of the *Leptin* gene in yellow cheek carp (*Elopichthys bambusa*), the full-length cDNA sequence of *leptin* was cloned, named *EbLep*. The full-length cDNA of *Eblep* was 1140 bp, and the length of the open reading frame (ORF), which can encode a protein of 174 amino acids, was 525 bp. The signal peptide was predicted to contain 33 amino acids. Sequence alignment showed that the amino acid sequence of *Leptin* was conserved in cyprinid fish. Despite large differences between primary structures, the tertiary structure of the EbLep protein was similar to that of the human protein and had four α-helices. The *EbLep* mRNA transcript was detected in all tested tissues, with the highest expression in the liver and lowest expression in the spleen. In this study, short-term fasting significantly increased the mRNA expression of *EbLep* in the liver, which returned to a normal level after 6 days of refeeding and was significantly lower than the normal level after 28 days of refeeding. In the brain, the mRNA expression of *EbLep* significantly decreased during short-term fasting and significantly increased to a higher value than the control group after 1 h of refeeding. It then rapidly decreased to a lower value than the control group after 6 h of refeeding, returning to the normal level after 1 day of refeeding, and significantly decreasing to a lower value than the control group after 28 days of refeeding. To sum up, the change in the mRNA expression of *EbLep* in the brain and liver may be an adaptive strategy for different energy levels.

## 1. Introduction

*Leptin* is a product of the obesity gene, which was identified by positional cloning technology [1]. *Leptin* is secreted by the adipose tissue in mammals and is considered an anorexia hormone [2,3,4]. The physiological role and regulatory mechanism of *leptin* have been extensively studied in many animals [5,6,7,8,9]. In mammals, *leptin* can reduce feeding and increase energy consumption through the regulation of feedback in the hypothalamic–pituitary axis [10]. In frogs (*Xenopus laevis*), *leptin* can influence limb growth and differentiation during early development [7]. In chickens, the expression of *leptin* and its receptor were detected in the brain and digestive tract [8], which suggests that *leptin* may be involved in brain and digestive tract-related functions. In *Anolis carolinensis*, *leptin* can ameliorate immunity [9]. In fish, *leptin* was first identified in *Takifugu rubripes* [11]. At present, *leptin* has been cloned from many species of fish, such as grass carp (*Ctenopharyngodon idella*), common carp (*Cyprinus carpio*), zebrafish (*Barchydanio rerio*), mandarin fish (*Siniperca chuatsi*), and orange-spotted grouper (*Epinephelus coioides*) [12,13,14,15,16]. Previous studies have shown that *leptin* plays a role in regulating food intake, glucose and lipid metabolism, reproduction, and immunity in fish [12,14,17,18,19,20]. Since teleost fishes exhibit a remarkable level of anatomical, ecological, behavioral, and genomic diversity, the structure and function of *leptin* may also vary considerably between fish species [21]. However, *leptin* from yellow cheek carp (*Elopichthys bambusa*) has not been cloned so far. Cloning of the *leptin* gene of yellow cheek carp is helpful to enrich the basic data of *leptin* in fishes, and provides reference material for further functional research on yellow cheek carp.

The liver is a central organ that controls metabolism in fish [22]. The liver, a main organ secreting *leptin* in fish, is first affected by different feeding states [18,23]. Several studies have shown that feeding status can affect the expression of *leptin* in fish [14,24] (Gambardella et al., 2012 and Gorissen et al., 2009). Gorissen et al., (2009) [14] confirmed that after fasting for a week, the mRNA level of *leptin-B* in the liver of zebrafish significantly decreased, while the mRNA level of *leptin-A* increased. In perch, the mRNA level of *leptin* in the liver significantly decreased after 3 weeks of starvation, and then increased after 3 weeks of feeding [25]. The regulation of food intake in fish was based on the integration of hypothalamic metabolic, endocrine, and circadian rhythm information [26], with mechanisms comparable but not identical [27] to those known in mammals [21]. The function of *leptin* in fish may be more complex than in mammals. There are few studies on the expression of *leptin* in the brain under different energy states in fish. More extensive and in-depth studies are needed to clarify the function and mechanism of *leptin* in the regulation of feeding status and energy metabolism.

Yellow cheek carp (*Elopichthys bambusa*), a member of the Cyprinidae and Leuciscinae, is a fierce carnivorous fish, which is mainly distributed in the plain area of the middle and lower Yangtze River. Known as the “freshwater tuna”, it has fresh and tender meat and a beautiful taste, high protein content, and a low fat and cholesterol content [28]. In China, managers of traditional fisheries are controlling populations of fierce carnivorous fish to reduce the threat to other fish larvae [29]. As a result, the population of fierce carnivorous fish, including the yellow cheek carp, has dramatically declined in natural water bodies. With the adjustment of aquaculture industry structure and the implementation of the “ten-year fishing ban” policy in China, the price of yellow cheek carp has continued to rise in recent years. However, basic research on the yellow cheek carp is particularly scarce. In the present study, the *Leptin* gene of yellow cheek carp has been cloned and its distribution patterns have been identified by analyzing the tissue expression of *EbLep*. Additionally, the changes in *Eblep* mRNA expression in the liver and brain during short-term fasting and refeeding has been studied. This study aimed to enrich the research conducted on the yellow cheek carp thus far and provide information for studying the function of *leptin* and its role in food intake and energy metabolism regulation.

## 2. Materials and Methods

### 2.1. Animals and Samples

The yellow cheek carp were provided by the Hunan Fisheries Research Institute and were obtained from the same parent group. A total of 390 healthy and neatly sized *Elopichthys bambusa*, with an average body weight of (221.36 ± 6.75 g) and average body length of (33.09 ± 1.33 cm), were selected from the pond. They were randomly and equally assigned to 6 cement pools (10 m × 5 m × 1 m) to acclimatize for 1 week. Commercial feed (crude protein ≥ 48%, crude fat ≥ 5.0%, lysine ≥ 2.8%, moisture ≤ 10%, ash ≤ 18%) was used twice a day (8:00 and 18:00) in all groups during the experimental period.

Six fishes were randomly collected from the cement pools and dissected after anesthesia with MS222. The liver, intestine, spleen, kidney, heart, gill, brain, head kidney, skin, and muscle were collected for cloning and the tissue expression analysis of *leptin*. Samples were quickly frozen in liquid nitrogen and then stored at −80 °C. 

In order to study the changes in the expression of *leptin* in the liver and brain under fasting and refeeding conditions, a control group and a treatment group were set up. There were three replicates in each group. The control group was fed continuously for 36 days, and the treatment group was fed for 28 days after 8 days of fasting. The control group and the treatment group were fed twice a day (8:00 and 18:00). Six *Elopichthys bambusa* were randomly collected from each group after fasting for 3 d (F3) and 8 d (F8), and were refed for 1 h (F8R1h), 6 h (F8R6h), 1 d (F8R1d), 6 d (F8R6d), and 28 d (F8R28d), respectively. Additionally, the liver and brain were sampled after anesthesia with MS-222. Samples were quickly frozen in liquid nitrogen and stored at −80 °C. During the experiment, the water temperature was (23.5 ± 3.4) °C, the pH was 6.7–7.2, and the dissolved oxygen was 5.7–6.5 mg/L.

### 2.2. Cloning of EbLep Gene

Liver RNA was extracted using Trizol, and first-strand cDNA was synthesized by reverse transcription using the RevertAid First Strand cDNA Synthesis Kit (Fermentas, Waltham, MA, USA). The quantity and quality of RNA were detected by Eppendorf (Hamburg, Germany) BioPhotometer Plus spectrophotometry. The ratio of absorbance at 260 and 280 nm (A260/A280) for samples was ranged from 1.8 to 2.0. According to the *leptin* sequence of grass carp (GenBank accession numbers FJ373293.1), the degenerate primer LEP-1 (Table 1) was designed by clustal X alignment software, version 2.1. The 50 μL reaction system contained PCR-grade water at 15.0 μL, 2X Ex taq buffer (Takara, Kusatsu, Japan) at 25.0 μL, dNTP mix (10 mM) at 1.0 μL, Ex taq (Takara) at 1.0 μL, cDNA first strand at 5.0 μL, primer F at 1.5 μL, and primer R at 1.5 μL. Reaction program: pre-denaturation at 94 °C for 2 min, denaturation at 94 °C for 30 s, annealing at 55 °C for 30 s, extension at 72 °C for 1 min, 35 cycles, and finally extension at 72 °C for 10 min. The PCR products were detected by 1% agarose gel electrophoresis, and the target fragments were purified using Gel Extraction Kit (OMEGA, Norcross, GA, USA). Then, the purified PCR products were cloned, and positive clones were screened for sequencing.

According to the core fragment sequence, the PCR primers for 5′-and 3′-RACE were designed (Table 1). To obtain the complete cDNA sequence of the *Eblep* gene, the 5′- and 3′-RACE PCRs were carried out using SMARTer RACE 5′/3′ Kit (Code No. 634858/59, Takara) by following the manufacturer’s instructions. The target product was cut from gel for purification. The purified PCR product was ligated with pMD18T, and the positive clones were sequenced.

According to the intermediate fragment sequence 5′RACE and 3′RACE results, the full-length cDNA sequence of *leptin* gene was spliced out. The gene start and stop codon positions were predicted by NCBI alignment analysis.

### 2.3. Sequence Analysis and Phylogenetic Tree Construction

The software DNAMAN was used to translate the sequence of the *leptin* gene to obtain the amino acid sequence. The amino acid sequences of *leptin* of other fishes and vertebrates were obtained from NCBI, and the multiple sequence alignment was performed using the software DNAMAN. The GenBank accession numbers of the sequences used in the figure were as follows: *Ctenopharyngodon idella* LEP, ACI32423.1; *Culter alburnus* LEP, AHI13615.1; *Megalobrama amblycephala* LEP, XP_048024644.1; *Carassius gibelio* LEP-A, ULE27184.1; *Cyprinus carpio* LEP-A2, AGK24956.1; *Labeo rohita* LEP, KAI2653424.1; *Schizothorax prenanti* LEP, AIE45855.1; *Schizothorax richardsonii* LEP, UNO36740.1; *Danio rerio* LEP-A, CAP47064.1; *Tachysurus fulvidraco* LEP, AFO67938.1; *Tachysurus vachellii* LEP, UNW38763.1; *Pygocentrus nattereri* LEP-A, XP_037396125.1; *Myxocyprinus asiaticus* LEP-A, XP_051580568.1; *Oreochromis niloticus* LEP-A, AHL37667.1; *Silurus meridionalis* LEP-A, XP_046720980.1; *Acipenser ruthenus* LEP, RXM28554.1; *Acipenser dabryanus* LEP-A, QSZ40475.1; *Chanos chanos* LEP-A, QNG41929.1; *Alosa sapidissima* LEP-A, XP_041965310.1; *Salvelinus alpinus* LEP, BAH83535.1; *Oncorhynchus keta* LEP-A, XP_035653655.1; *Astyanax mexicanus* LEP-A, XP_022535129.1; *Colossoma macropomum* LEP-A, XP_036441558.1; *Xenopus laevis* LEP, XP_018108304.1; *Homo sapiens* LEP, AAH69452.1.

A protein phylogenetic tree was established based on amino acid sequences by the NJ method (neighbor joining) in MEGA 11.0. The number of bootstrap verifications was set to 1000. Signal peptides were predicted using signalP-5.0 (https://services.healthtech.dtu.dk/service.php?SignalP-5.0 accessed on 12 October 2022). The SWISS-MODEL (https://swissmodel.expasy.org/ accessed on 12 October 2022) modelling function was used to predict the secondary and tertiary structure of *leptin*. 

### 2.4. Tissue Distribution and Fasting–Refeeding Expression

The primer *q-leptin* was designed according to the cloned *leptin* gene cDNA sequence, and *β-actin* was selected as the reference gene [30] (Table 1). The molality of *q-leptin* F and R are 5.26 and 4.83 nmol/OD, and the molality of *β-actin* F and R are 6.37 and 5.89 nmol/OD. cDNA used for *leptin* gene quantification was synthesized using the PrimeScript™ RT reagent Kit with the gDNA Eraser kit. 

Real-time PCR assays were carried out to examine the distribution of the *leptin* gene in various tissues—including the liver, intestine, spleen, kidney, heart, gill, brain, head kidney, skin, and muscle, and the expressions of the brain and liver under different feeding conditions—with 12.5 μL reaction volume containing 6 μL of SYBR Premix Ex TaqTM II, 1 μL of cDNA obtained by reverse transcription, 4.5 μL of RNase-free water, 0.5 μL of upstream primer, and 0.5 μL of downstream primer. The reaction was pre-denatured at 95 °C for 3 min, denatured at 95 °C for 5 s, annealed at 60 °C, and extended for 20 s for 39 cycles. Three technical replicates were used for each set of reactions. All operations are carried out according to the kit instructions.

### 2.5. Data Analysis

The relative expression results were calculated using the formula R = 2^−ΔΔCt^. All data were shown as mean ± standard deviation (SD) and were subjected to the one-way ANOVA using the software SPSS 18.0, with *p* < 0.05 as a significant difference. After the one-way ANOVA, the homogeneity-of-variance was conducted, and when the Sig value was greater than 0.05, the variance could be considered homogeneous.

## 3. Results

### 3.1. Characterization of EbLep

**Figure 1:**
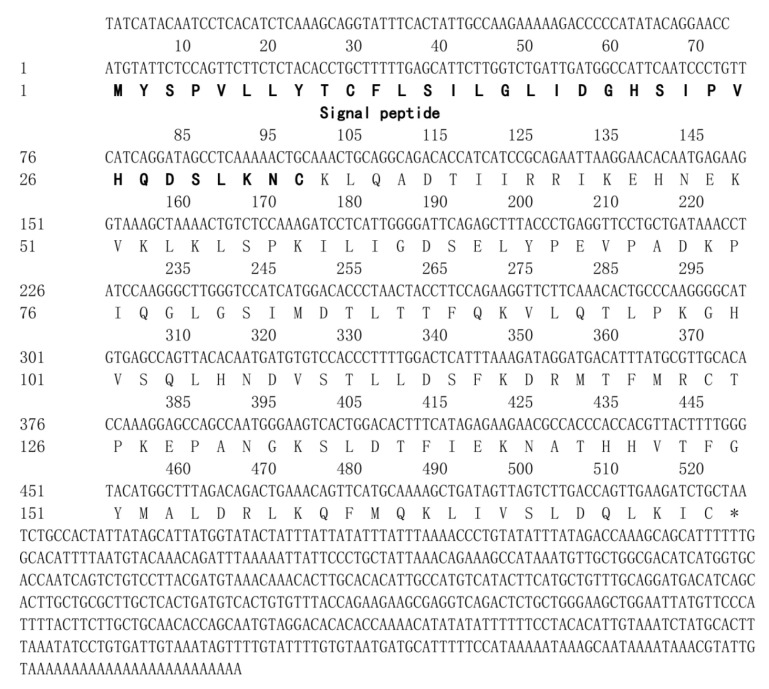
Nucleotide sequence and deduced amino acid sequence of the *leptin* gene of Elopichthys bambusa. The sense strand is displayed from the 5′ to 3′ direction. Stop codons are marked with “*” in the nucleotide sequence. Signal peptides are marked in bold letters. The GenBank accession number is MW794324.

The full-length cDNA sequence of *leptin* gene of yellow cheek carp was obtained by RT-PCR and RACE (GenBank Accession No. MW794324). The full-length cDNA of *leptin* is 1140 bp, and the length of the open reading frame (ORF) is 525 bp, which can encode a protein of 174 amino acids. The signal peptide was predicted to contain 33 amino acids by NCBI BLAST (Figure 1).

**Figure 2:**
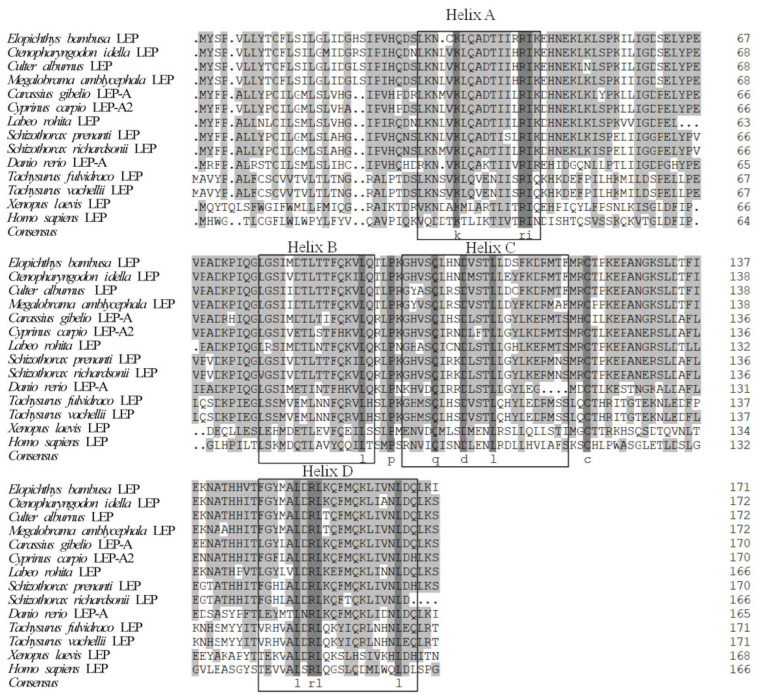
Amino acid sequence alignment among *EbLep* and *leptin* of other vertebrates. Omitted portions are indicated by dots, and shaded areas indicate residues that are 100% shared by all sequences. Four α-helices are framed.

Sequence alignment shows that the amino acid sequence of *leptin* was conserved in cyprinid fish, such as yellow cheek carp (*Elopichthys bambusa*), grass carp (*Ctenopharyngodon idella*), *Megalobrama amblycephaloid*, common carp (*Cyprinus carpio*), and crucian carp (*Carassius gibelio*), but had a large difference from the sequences of non-cynic fishes and other vertebrates. However, the secondary structure of *leptin* proteins from various animals was highly conserved, with four α-helices (Helix A–D) (Figure 2)

The amino acid sequence conservation of *leptin* was low between the yellow cheek carp and human, with only 23.20% sequence identity. The amino acid sequence identity of *EbLep* and other fishes was *Ctenopharyngodon idella* (91.60%), *Culter alburnus* (91.60%), *Megalobrama amblycephala* (91.00%), *Carassius gibelio* (76.10%), *Cyprinus carpio* (76.80%), *Labeo rohita* (74.80%), *Schizothorax prenanti* (74.20%), *Schizothorax richardsonii* (74.20%), *Danio rerio* (60.00%), *Tachysurus fulvidraco* (40.00%), and *Tachysurus vachellii* (40.00%), respectively (Table 2).

**Figure 3:**
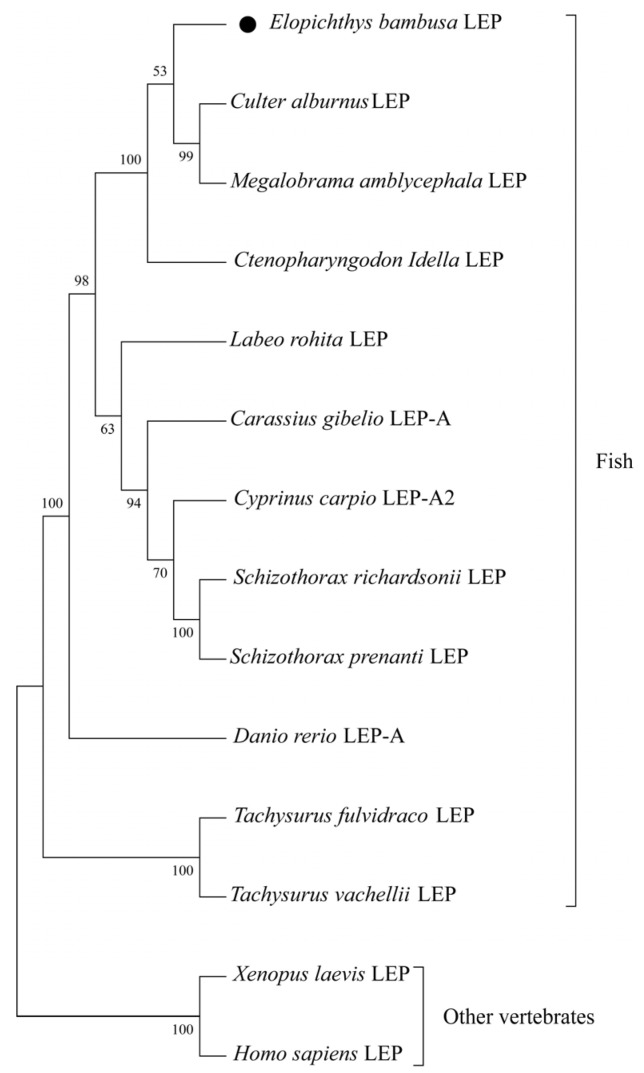
Phylogenetic tree based on amino acid sequence of *leptin*. The phylogenetic tree was established by the NJ method (neighbor-joining) in MEGA 11.0. The number of bootstraps verifications was set to 1000 times.

**Figure 4:**
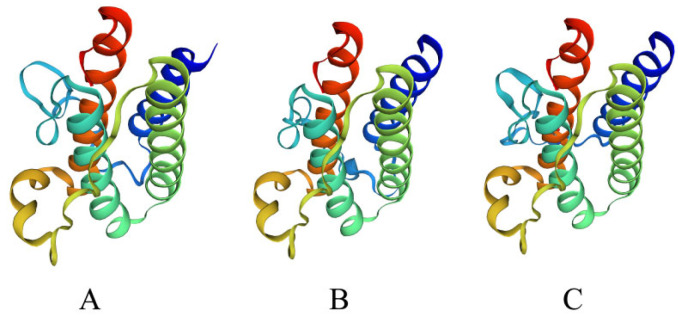
Tertiary structure model of *Leptin* predicted from amino acid sequences. (**A**) Yellow Cheek Carp, (**B**) Human, (**C**) Grass Carp.

The phylogenetic tree constructed based on the amino acid sequence of *leptin* is shown in Figure 3. All teleost fish LEPs are grouped into one clade, and two cartilaginous fishes (*Acipenser ruthenus* and *Acipenser dabryanus*) and other vertebrates (*Xenopus laevis* and *Homo sapiens*) LEPs are grouped into one clade. The closest to *EbLep* were *Culter alburnus* and *Megalobrama amblycephala*. Despite large differences between primary structures (Figure 2 and Table 2), the tertiary structure of EbLep protein was also predicted to be similar to that of humans (Figure 4).

### 3.2. Tissue Expression of EbLep

**Figure 5:**
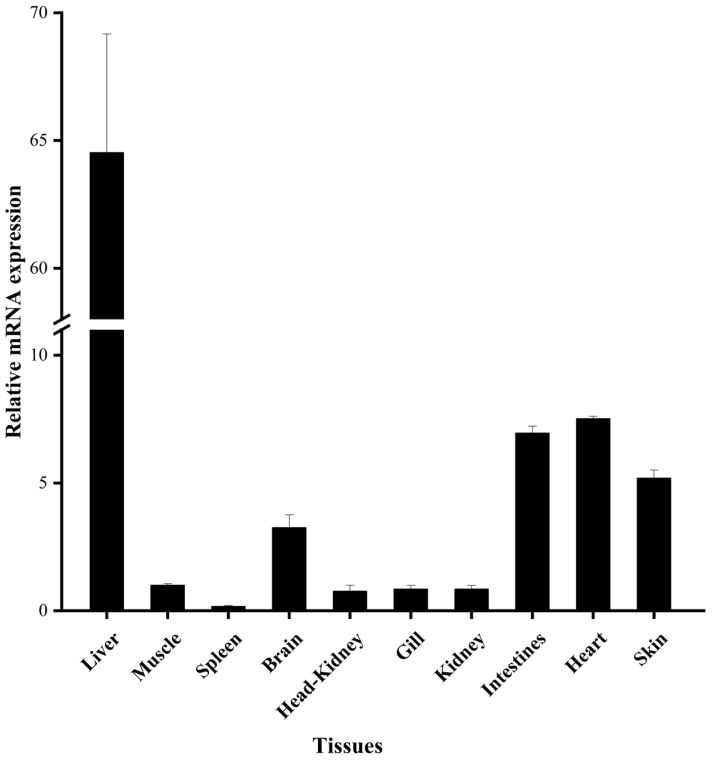
Relative mRNA expression of *EbLep* in various tissues. All values represent the mean ± S.D. (*n* = 6).

The mRNA tissue expression levels of *EbLep* were analyzed by real-time PCR. The results show that *EbLep* can be expressed in liver, intestine, spleen, kidney, heart, gill, brain, head kidney, skin, and muscle. The mRNA expression level in the liver tissue was the highest, which was more than eight times that of other tissues. This was followed by the heart, intestine, skin, brain, muscle, kidney, gill, head, kidney, and spleen (Figure 5).

### 3.3. Expression of EbLep mRNA in the Liver and Brain under Fasting and Refeeding

**Figure 6:**
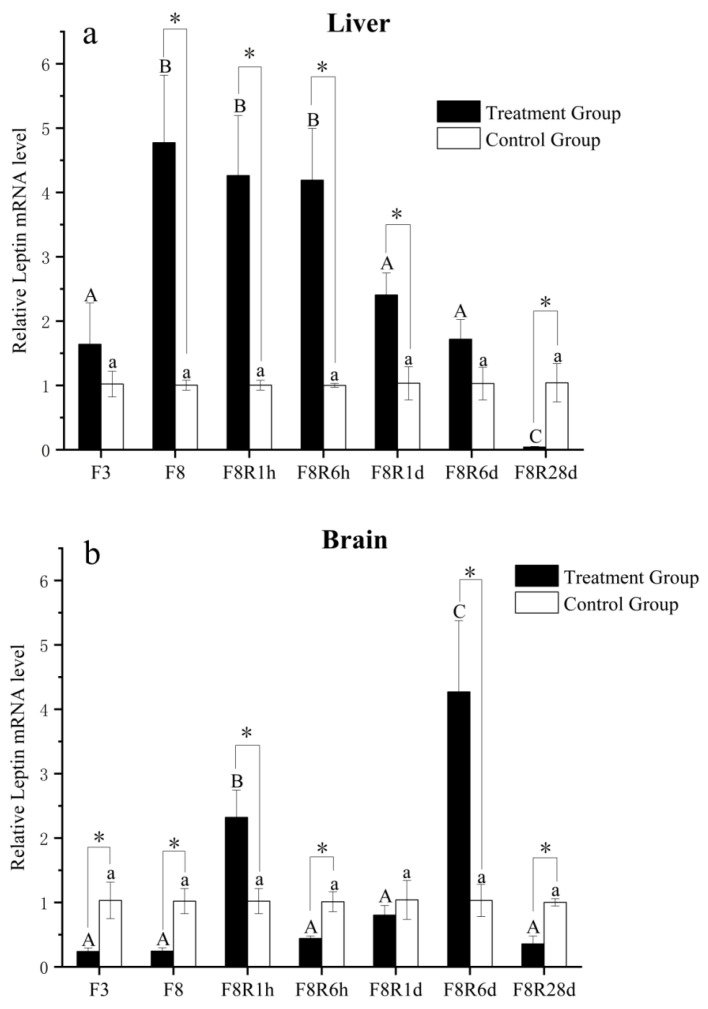
Relative Expression of *EbLep* mRNA in the liver (**a**) and brain (**b**) under fasting and refeeding. F3 and F8 indicate fasting for 3 and 8 days, respectively. F8R1h, F8R6h, F8R1d, F8R6d, and F8R28 indicate refeeding for 1 h, 6 h, 1 day, 6 days, and 28 days after 8 days of fasting, respectively. Significant differences among treatment groups (ANOVA, *p* < 0.05) are indicated by different capital letters (A, B, and C); no significant differences among control groups (ANOVA, *p* > 0.05) are indicated by different lowercase letters (a); and significant differences between control and treatment groups (ANOVA, *p* < 0.05) are indicated by *.

The *EbLep* mRNA expression in the liver increased after fasting, as determined by real-time PCR (Figure 6). Compared with the control group, there was no significant difference after starvation for 3 days (*p* > 0.05), but there was a significant difference after starvation for 8 days (*p* < 0.05). After refeeding, the expression of *EbLep* mRNA in the liver showed a downward trend. One day after refeeding, it showed a significant decrease (*p* < 0.05), and 28 days after refeeding, it was significantly lower than the control group (*p* < 0.05) (Figure 6a).

Fasting significantly decreased the expression of *EbLep* mRNA in the brain. After refeeding for 1 h, the expression of *EbLep* mRNA in the brain of the treatment group rapidly increased and became significantly higher than the control group (*p* < 0.05). After 6 h of refeeding, it gradually decreased and became significantly lower than that of the control group (*p* < 0.05). The expression of *EbLep* mRNA in the brain of the treatment group significantly increased and reached the highest level at 6 days of refeeding, followed by a significant decrease at 28 days (*p* < 0.05) (Figure 6b).

## 4. Discussion

Johnson et al., (2000) [31] first detected the presence of *leptin* in the blood, brain, heart, and liver of sunfish (*Lepomis macrochirus*), rainbow trout (*Oncorhynchus mykiss*), largemouth bass (*Pomonix annularis*), and microbleeker (*Ictalurus punctatus*). The *leptin* gene was later confirmed to exist in many other fish species [11,32,33]. *Leptin* in fish generally exists in two subtypes, named *lep-a* and *lep-b* [14,34]. This may be related to the fact that genome duplication occurred in the evolutionary process of fish [35]. The two isoforms encode two different products with low amino-acid identity (20–30%) [36]. In this study, the *Leptin* gene (*EbLep*) identified from the yellow cheek maybe was maybe lep-a by NCBI blast, but it cannot be determined whether other copies of this gene are present in the yellow cheek carp genome. 

The yellow cheek carp shares only 23.20% sequence identity with human *leptin*. Similarly, goldfish (*Carassius auratus*) [37] and mandarin fish (*Siniperca chuatsi*) [15] have very low amino acid sequence homologies with mammalian *leptin*, both of which are less than 30%. This reflects the high evolutionary rate of the *leptin* gene. The amino acid sequences were quite different from those of other mammals [21]. The nucleic acid and amino acid sequences of *leptin* greatly differ from different fish species, but the tertiary structure of its protein is very conserved. They are highly similar in their predicted tertiary structure, when modelled based on the crystal structure of human *leptin* [11,38,39]. Our finding was in accordance with recent studies indicating that the tertiary structure of proteins is important for the main physiological function of the *leptin* system in fish. Kurokawa and Murashita (2009) [34] also had similar conclusions.

*Leptin* is expressed in various tissues; mammalian *leptin* is mainly expressed in adipose tissue [40]. Unlike mammals, the brain and heart are the main synthetic organs in *Xenopus* [7]. The expression level of *leptin* from Kermani sheep was highest in the adipose tissue and liver and lowest in the heart [20]. *Leptin* is slightly expressed in different tissues, such as the gut, adipose tissue, and brain in teleost fishes, but is mainly distributed in the liver [15,37]. However, some fishes have a high expression of *leptin* in other tissues. The expression of *leptin* in the kidneys, gills, intestines, and gonads of *Megalobrama amblycephala* was much higher than in the liver [41]. The *leptin* of Atlantic salmon (*Salmo salar*) had the highest expression in the brain and muscle [39]. In this paper, the tissue expression level of *EbLep* was highest in the liver, medium in the heart, intestine, skin, and brain, and lowest in the muscle, spleen, kidneys, gills, head, and kidney. The liver is the metabolic energy center of fish. Our findings match those observed in earlier studies [15,37], suggesting that *EbLep* may be involved in the regulation of energy balance. At the same time, *leptin* is commonly expressed in other tissues of yellow cheek carp. This suggests that the *EbLep* may be involved in many other physiological processes, and further studies are required.

To understand the physiological function of *leptin* in the yellow cheek carp, we compared the mRNA expression of *EbLep* in the liver and brain under different feeding states (short-term fasting and refeeding). We found that short-term fasting significantly increased the mRNA expression of *EbLep* in the liver, which returned to normal level after 6 days of refeeding and was significantly lower than the normal level after 28 days of refeeding. Similar findings were found in orange-spotted grouper [16], zebrafish (*leptin-A*) [14] and rainbow trout [42], and the opposite results were found in common carp [38], *Acrossocheilus fasciatus* [43], striped bass [25], and mandarin fish [15]. Through a comparison, it was found that the fasting time in the studies with similar results to this paper was more than 3 days, while the fasting time of the opposite results was less than 1 day. Therefore, we hypothesized that if the fasting time was less than 1 day, the mRNA expression of *leptin* in the liver would be reduced to promote appetite, and if the starvation time was more than 3 days, the mRNA expression of *leptin* in liver would be increased, in turn boosting glycolipid catabolism to provide energy for normal activities. The above conclusion needs to be verified in further research. It is worth noting that there were significant differences in the expression of *leptin* in the liver under different feeding states in the above studies. This suggests that *leptin* may play an important role in regulating energy status. The results of most fish studies show that specific *leptin* has an inhibitory effect on feeding [12,17,44]. It was speculated that *leptin* regulates the balance of feeding by regulating energy status in fish. The mechanism of the mRNA expression of *leptin* in the liver, which is adapted to fasting and refeeding, requires further research.

In fish, the endocrine signals in the hypothalamus region that influence the brain’s regulation of food intake could be triggered by different nutritional and metabolic conditions [45,46]. Genes that control these endocrine signals (*leptin* is one of them) play an important role in appetite regulation [47]. Several studies have shown that *leptin* could inhibit food intake [12,17,44]. Murashita et al., (2008) [17] believed that *leptin* could regulate food intake by stimulating expression of the appetite inhibiting factor *proopiomelanocorein-A1/A2* (*POMC-A1/A2*) gene and reducing the expression level of the appetite stimulating factor *neuropeptide Y* (*NPY*) gene. At the same time, it has been reported that fasting could cause a downregulation of *leptin* gene expression in many aquatic animals and mammals [15,48,49]. In this study, the mRNA expression of *EbLep* in the brain was also significantly decreased during short-term fasting. Therefore, we thought that the downregulation of *leptin* caused by fasting may be related to the regulation of appetite in fish. Yuan et al., (2014) [50] had a similar view. In mammals, *leptin* could regulate feeding behavior by controlling the expression of some anorexia genes in the brain (e.g., *Cart*, *Crh*, *Mc4r*, *POMC*) [51,52,53]. In fish, regulation of feeding homeostasis is based on the hypothalamus’ integration of metabolic and endocrine information [26]. This suggests that *EbLep* might regulate hypothalamic neuropeptides to regulate appetite during the fasting state. In this paper, the mRNA expression of *EbLep* in the brain significantly increased to higher than that in the control group after 1 h refeeding, and rapidly decreased to lower than that in the control group after 6 h of refeeding. My previous study found that the intestinal contents were less than 30% after 6 h of feeding [54]. This suggests that the *EbLep* expression in the brain may be related to the amount of food in the gut. In addition, the expression of *EbLep* in the brain returned to a normal level after 1 day of refeeding, and significantly decreased to a lower value than the control group after 28 days of refeeding. Several studies also thought that energy status could regulate food intake by regulating *leptin* expression in brain [55,56]. Therefore, the results of this study may be caused by the difference in energy status between 1 h, 6 h, 1 d, and 28 d after refeeding. In summary, *EbLep* expression in the brain may be related to the regulation of appetite. The change in the mRNA expression of *leptin* in the brain of yellow cheek carp may be an adaptive strategy for different energy levels.

## 5. Conclusions

In this study, we cloned the full-length cDNA sequence of *Eblep*, which was 1140 bp; the length of the open reading frame (ORF), which can encode a protein of 174 amino acids, was 525 bp. The signal peptide was predicted to contain 33 amino acids. The *EbLep* mRNA transcript was detected in all tested tissues with the highest expression in the liver and lowest expression in the spleen. By studying the expression of *EbLep* mRNA in the liver and brain under fasting and refeeding, we found that the change in the mRNA expression of EbLep may be an adaptive strategy for different energy levels.

## Figures and Tables

**Table 1:** Primer sequences for PCR.

Purpose	Name	Sequence (5′-3′)	Annealing Temperature	E-Values
Core Fragment Sequence Acquisition	LEP-1	F: ATGTATTYTCCAGYTCTTC	55 °C	
		R: GCATGAACTSTKTCAGTC		
5′-RACE PCR	LEP-5′-RACE-1	ATGATGGTGTCTGCCT		
	LEP-5′-RACE-2	TGAGGCTATCCTGATG		
	LEP-5′-RACE-3	CCATCAATCAGACCAAGAAT		
3′-RACE PCR	LEP-3′-RACE-1	CCAAGGGGCATGTGAGCCAGTTAC		
	LEP-3′-RACE-2	GCACACCAAAGGAGCCAGCCAATG		
Expression Examination	*q-leptin*	F: ACTGTCTCCAAAGATCCTCA	60 °C	100.3%
		R: AAAAGGGTGGACACATCATT		
Reference Genes	*β-actin*	F: CCTGTATGCCTCTGGTCG	60 °C	98.6%
		R: CTCGGCTGTGGTGGTGAA		

Note: Primer synthesis and sequencing in this experiment were performed by Sangon Biotech (Shanghai) Co., Ltd.

**Table 2:** Amino acid sequence identities of EbLep compared with *leptin* of various vertebrates.

	Eb LEP	Ci LEP	Ca LEP	Ma LEP	Cg LEP-A	CcLEP-A2	Lr LEP	Sp LEP	Sr LEP	Dr LEP-A	Tf LEP	Tv LEP	Xl LEP	Hs LEP
Eb LEP	100.00%													
Ci LEP	91.60%	100.00%												
Ca LEP	91.60%	89.70%	100.00%											
Ma LEP	91.00%	89.70%	96.10%	100.00%										
Cg LEP-A	76.10%	75.50%	72.90%	73.50%	100.00%									
Cc LEP-A2	76.80%	76.80%	74.20%	74.20%	87.10%	100.00%								
Lr LEP	74.80%	73.40%	72.90%	71.60%	78.80%	77.40%	100.00%							
Sp LEP	74.20%	74.20%	72.30%	73.50%	83.20%	86.50%	75.50%	100.00%						
Sr LEP	74.20%	72.90%	72.30%	73.50%	83.20%	86.50%	75.50%	97.40%	100.00%					
Dr LEP-A	60.00%	59.40%	58.70%	59.40%	63.90%	65.20%	63.90%	61.90%	61.90%	100.00%				
Tf LEP	40.00%	40.00%	40.00%	40.60%	38.70%	41.30%	37.40%	40.60%	41.30%	36.80%	100.00%			
Tv LEP	40.00%	40.00%	40.00%	40.60%	39.40%	41.90%	38.10%	41.30%	41.90%	37.40%	99.40%	100.00%		
Xl LEP	27.70%	30.30%	28.40%	28.40%	28.40%	26.50%	27.10%	27.10%	26.50%	27.70%	29.00%	29.00%	100.00%	
Hs LEP	23.20%	22.60%	21.30%	21.30%	22.60%	25.20%	23.90%	25.20%	23.90%	23.20%	21.30%	21.30%	35.50%	100.00%

Note: The abbreviations of the species listed in this table are as follows: *Elopichthys bambusa* (Eb); *Ctenopharyngodon idella* (Ci); *Culter alburnus* (Ca); *Megalobrama amblycephala* (Ma); *Carassius gibelio* (Cg); *Cyprinus carpio* (Cc); *Labeo rohita* (Lr); *Schizothorax prenanti* (Sp); *Schizothorax richardsonii* (Sr); *Danio rerio* (Dr); *Tachysurus fulvidraco* (Tf); *Tachysurus vachellii* (Tv); *Xenopus laevis* (Xl); *Homo sapiens* (Hs).

## Data Availability

The datasets generated and analyzed during the current study are available in the National Center for Biotechnology Information, USA National Library of Medicine repository, National Center for Biotechnology Information (nih.gov, accessed on 19 April 2023). The sequence of *Eblep* was submitted to the NCBI with GenBank Accession No. MW794324.
